# Novel Model of Tendon Regeneration Reveals Distinct Cell Mechanisms Underlying Regenerative and Fibrotic Tendon Healing

**DOI:** 10.1038/srep45238

**Published:** 2017-03-23

**Authors:** Kristen Howell, Chun Chien, Rebecca Bell, Damien Laudier, Sara F. Tufa, Douglas R. Keene, Nelly Andarawis-Puri, Alice H. Huang

**Affiliations:** 1 Dept. of Orthopaedics, Icahn School of Medicine at Mount Sinai, New York, NY 10029 USA; 2 Dept. of Mechanical and Aerospace Engineering, Cornell University, Ithaca, NY 14853 USA; 3 Micro-Imaging Center, Shriners Hospital for Children, Portland, OR 97209, USA

## Abstract

To date, the cell and molecular mechanisms regulating tendon healing are poorly understood. Here, we establish a novel model of tendon regeneration using neonatal mice and show that neonates heal via formation of a ‘neo-tendon’ that differentiates along the tendon specific lineage with functional restoration of gait and mechanical properties. In contrast, adults heal via fibrovascular scar, aberrant differentiation toward cartilage and bone, with persistently impaired function. Lineage tracing identified intrinsic recruitment of *Scx-lineage* cells as a key cellular mechanism of neonatal healing that is absent in adults. Instead, adult *Scx-lineage* tenocytes are not recruited into the defect but transdifferentiate into ectopic cartilage; in the absence of tenogenic cells, extrinsic αSMA-expressing cells persist to form a permanent scar. Collectively, these results establish an exciting model of tendon regeneration and uncover a novel cellular mechanism underlying regenerative vs non-regenerative tendon healing.

Tendons are dense connective tissues that mediate transfer of muscle forces to the skeleton. This important mechanical function is enabled by a highly organized extracellular matrix primarily composed of aligned type I collagen fibers. With acute injury or tendinopathy, tendon function is often permanently compromised due to poor healing and scarring (defined as disorganized fibrovascular matrix and inferior mechanical properties), leading to chronic pain and prolonged disability^1^. Despite the high incidence of injures (tendon and ligament injuries affect 110 million patients in the US alone)^2^, treatment options remain few with variable success outcomes.

To date, the cell and molecular mechanisms that drive tendon differentiation and maturation remain poorly understood. The key transcription factors and signaling pathways identified for tendon were originally discovered from studies of embryonic development^3^,^4^,^5^,^6^. Of these transcription factors, *Scleraxis (Scx*), is still the earliest known marker expressed by tendon progenitors, while *Mohawk (Mkx*) is a key regulator of postnatal collagen maturation^7^,^8^,^9^,^10^. While significant progress has been made in understanding tendon development, the biological events that orchestrate tendon differentiation, maturation, and scar formation in the context of healing are still largely unknown.

Most of the existing research in tendon healing has been carried out using adult animal models that heal via the default pathway of fibrovascular scar. However, the cells that regulate fibrotic tendon healing have not been fully defined since lineage tracing studies in this context are few. The paucity of tendon regeneration models is also a major limitation, since regenerative healing will likely involve cellular players, signaling pathways, and unique mechanical and immune environments that are absent during development. The neonatal mouse recently emerged as an exciting model of mammalian regeneration for the heart, which heals by scar in the adult^11^. A few studies in other tissue systems (cochlear hair cells and digit tips) suggest that this neonatal regenerative capacity may extend to a wide range of tissues, although this remains unconfirmed for most tissues^12^,^13^,^14^,^15^. Although one intriguing study for tendon showed restoration of mechanical properties one week after neonatal injury, the cells that mediate healing were not determined^16^. In this study, we address this limitation in the field and show for the first time that neonatal tendon has regenerative capacity, with full restoration of function and tendon-specific differentiation after injury. Using genetic tools, we further show that tendon regeneration is driven by two cell populations: 1) an early population of extrinsic αSMA cells that are transiently recruited and 2) intrinsic tenocytes that proliferate and differentiate to regenerate the tendon. In adults, tendon cells undergo aberrant differentiation toward cartilage and are not recruited into the defect; in the absence of tenogenic cells, extrinsic fibrotic cells persist to form a permanent scar. Collectively, these results establish exciting models of regenerative and non-regenerative tendon healing in a genetically tractable background, and define a potential cellular mechanism for improving adult tendon healing.

## Results

### Neonatal tendon heals via regeneration of *ScxGFP*+ ‘neo-tendon’

**Figure 1:**
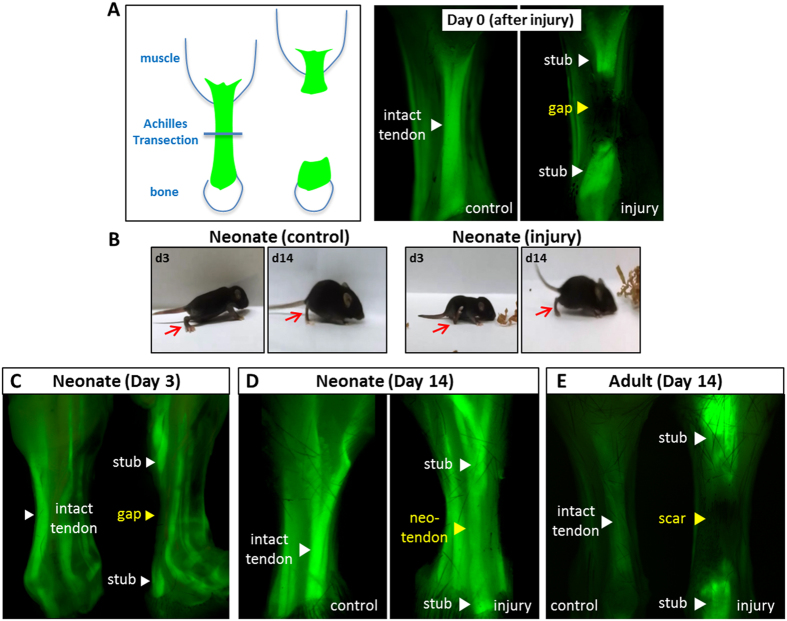
Neonatal tendon heals via formation of *ScxGFP*+ neo-tendon while adult tendon heals via *ScxGFP*-negative scar. (**A**) Schematic and whole mount images of Achilles tendon transection injury model using *ScxGFP* mice. (**B**) Video still frames of neonatal mice at d3 and d14 after P5 injury. Complete videos can be found in Supplementary Data. Red arrows indicate injured limb and comparable control limb in non-injured animals to highlight abnormal gait. Whole mount images of control and injured *ScxGFP* limbs at (**C**) d3 and (**D**) d14 after neonatal injury. White triangles indicate intact tendon and transected tendon stubs. Yellow triangles indicate gap space at d3 and *ScxGFP*+ neo-tendon at d14. (**E**) Whole mount images of control and injured *ScxGFP* limbs at d14 after adult injury. White triangles indicate intact tendon and transected tendon stubs while yellow triangle indicates *ScxGFP*-negative scar tissue.

To test whether neonatal tendons are capable of regeneration, we used a simple transection injury. For all animals, Achilles tendon transection resulted in immediate retraction of the divided tendon stubs leaving an empty gap space in between (Fig. 1A). At day 3 (d3) after tendon transection, the injured limb in neonates appeared drastically impaired; pups were unable to maintain normal posture on the injured limb and ambulation was achieved predominantly by weight bearing on the injured heel/ankle instead of toes (Fig. 1B and Supplementary Videos S1,S2,S3,S4). By d14 however, neonatal mice were fully mobile and qualitative differences between injured and uninjured limbs were no longer obvious (Fig. 1B).

At the tissue level, whole mount imaging of *ScxGFP* hindlimbs at d3 showed that *ScxGFP* expression was restricted to the original tendon stubs and that the gap space was devoid of *ScxGFP*+ cells. By d14 in the neonatal group, a continuous and aligned *ScxGFP*+‘neo-tendon’ had formed within the gap space, connecting the original tendon stubs, suggesting that rapid regeneration of tendon may be possible in neonates (Fig. 1C,D). In adult limbs, we found that *ScxGFP* expression was downregulated in tendon following skeletal maturity, although low expression was still detectable relative to non-tendon tissues. Surprisingly, at d14 after adult injury, dramatic re-activation of *ScxGFP* was observed, but only in the original tendon stubs (non-injured tendons adjacent to the Achilles were not affected) (Fig. 1E). Although the gap space was also filled by new tissue at d14 in adults, this tissue did not express *ScxGFP*. Thus, in contrast to our findings in neonates, tendon transection injury in adult tendons healed via formation of *ScxGFP*-negative tissue bridging the tendon stubs, suggesting there are distinct cellular mechanisms that regulate neonatal vs adult tendon healing.

### Tendon-specific differentiation during neonatal healing and neo-tendon formation

**Figure 2:**
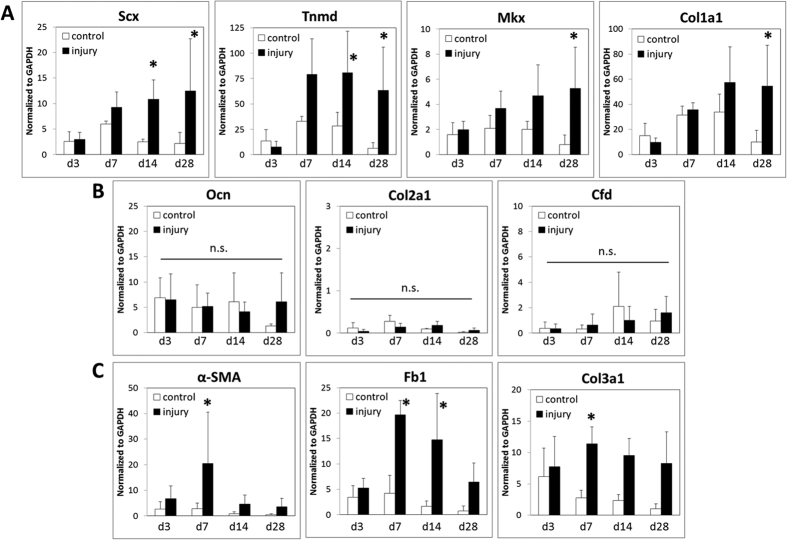
Expression of tendon-specific genes during neonatal regeneration. Real time qPCR of (**A**) tendon markers, (**B**) cartilage, bone, and fat markers, and (**C**) scar-associated markers in control and injured neonatal tendons. *Indicates significant difference relative to control within timepoint (p < 0.05). n.s. indicates no significance relative to control (p > 0.1). n = 5–7 tendons/group.

To test whether neonatal healing is tendon-specific, we evaluated the expression of several markers associated with tendon, cartilage, bone, or fat differentiation from d3 thru d28 after injury. Real time qPCR showed enhanced expression of all tendon markers after neonatal injury, with significant differences in tendon differentiation markers *Scx* and *Tnmd* at d14 and d28 (p < 0.05 vs control) (Fig. 2A). Interestingly, tendon markers associated with collagen fibrillogenesis, such as *Mkx* and *Col1a1,* were not significantly upregulated until d28 (p < 0.05 vs control) (Fig. 2A). Overall, while control samples did not vary across timepoints for any of the genes assayed, injured samples increased expression over time (p < 0.05 d3 vs d28).

Markers for osteogenesis (*Ocn*), chondrogenesis (*Col2a1*), and adipogenesis (Cfd) were not significantly upregulated after injury at any timepoint investigated (Fig. 2B). Transient upregulation of scar-associated markers (*α-SMA, Fb1,* and *Col3a1*) was observed, beginning at d7, before returning to control levels by d28 (Fig. 2C). Finally, analysis of adult tendons showed that tendon markers were not significantly upregulated after injury at any timepoint. Indeed, we found down-regulation of *Scx* (trend, p = 0.076) and *Mkx* (p < 0.05) at d14 compared to control (Table S1). Collectively, these results show that neonatal tendon regeneration progresses through transient expression of fibrotic markers followed by tendon-specific differentiation. Our results also show that aberrant differentiation toward alternative mesenchymal lineages does not occur during neonatal healing.

### Functional gait and mechanical properties are restored during neonatal tendon healing

**Figure 3:**
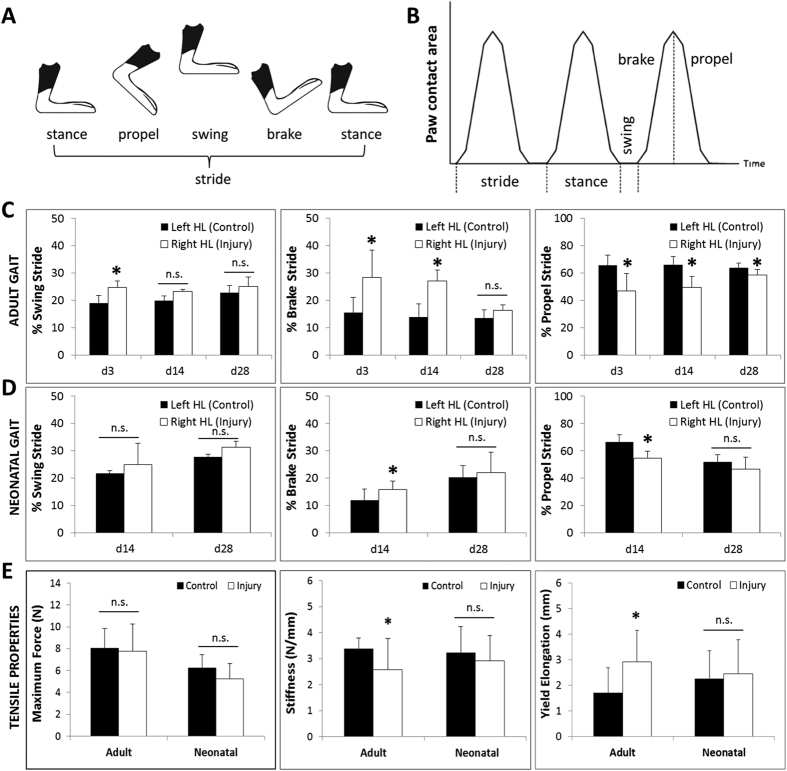
Full recovery of functional properties after neonatal tendon injury. (**A**) Schematic and (**B**) idealized graph defining gait parameter measurements (adapted from Digigait Imaging Systems). (**C**) Adult gait parameters tested from d3 to d28 after injury. (**D**) Neonatal gait parameters tested at d14 and d28 after injury. *Indicates significant difference relative to control limb within timepoint. n = 5 mice/group. (**E**) Tensile properties maximum force, stiffness, and yield elongation at d56 after adult and neonatal injury. *Indicates significant difference relative to contralateral control (p < 0.05). n.s. indicates no significance relative to control (p > 0.1). n = 10 tendons/group.

To determine whether functional properties are restored after tendon injury, we quantified hindlimb gait and tendon mechanical properties (Fig. 3). To identify reproducible parameters associated with injury, we evaluated adult injured mice 3 days after injury. The d3 timepoint was chosen since both neonates and adults were visibly impaired at this time; we focused on adults since neonates at this timepoint (P8) were still too immature to walk consistently on the treadmill. Comparing non-injured control animals and injured animals (in which the right Achilles tendon was transected), we identified three parameters that were significantly different in injured limbs: %Swing Stride, %Brake Stride, and %Propel Stride (since male and female mice were used, all parameters were normalized by Stride length to minimize differences due to animal size/age). The Achilles tendon primarily functions in planar flexion, which regulates the propulsive or ‘lift-off’ phase of gait; thus %Propel Stride is the parameter most specific for Achilles function. For all three parameters, the injured (right) hindlimb was significantly impaired compared to the contralateral control (left) hindlimb (p < 0.05; Fig. 3C). While %Swing Stride was recovered in both adult and neonatal injury groups by day 14, %Brake Stride and %Propel Stride remained significantly abnormal (p < 0.05, Fig. 3C,D). However, the difference between left and right hindlimbs was already much reduced in the injured neonatal group compared to adult for both parameters, indicating more rapid recovery (neonate vs adult; %Brake Stride: 25% vs 50% diff; %Propel Stride: 17% vs 25% diff). By day 28, all gait parameters were normal in the injured neonatal group, while %Propel Stride remained significantly abnormal for the adult injured group (p < 0.05, Fig. 3C,D). Note that no differences were ever detected between left and right hindlimbs of non-injured, age-matched control mice at any timepoint (p > 0.1; Supplementary Fig. S1).

As an additional test for tendon function, we also carried out direct mechanical testing to determine tensile properties at d56 after injury (Fig. 3E). Consistent with previous reports in the literature, adult tendons did not fully regain mechanical properties after injury. While maximum force was recovered for both groups, we found that the tensile stiffness of adult tendon remained significantly lower after injury compared to the contralateral control tendon; elongation at yield was also abnormal (p < 0.05; Fig. 3E). Strikingly, the tensile properties of neonatal injured tendons were all fully restored by d56 (p > 0.1, Fig. 3E). Taken together, this data suggests that neonatal tendons heal more rapidly compared to adult tendons, with complete restoration of functional properties.

### Extracellular matrix composition and collagen fibril ultrastructure is improved after neonatal injury compared to adults

**Figure 4:**
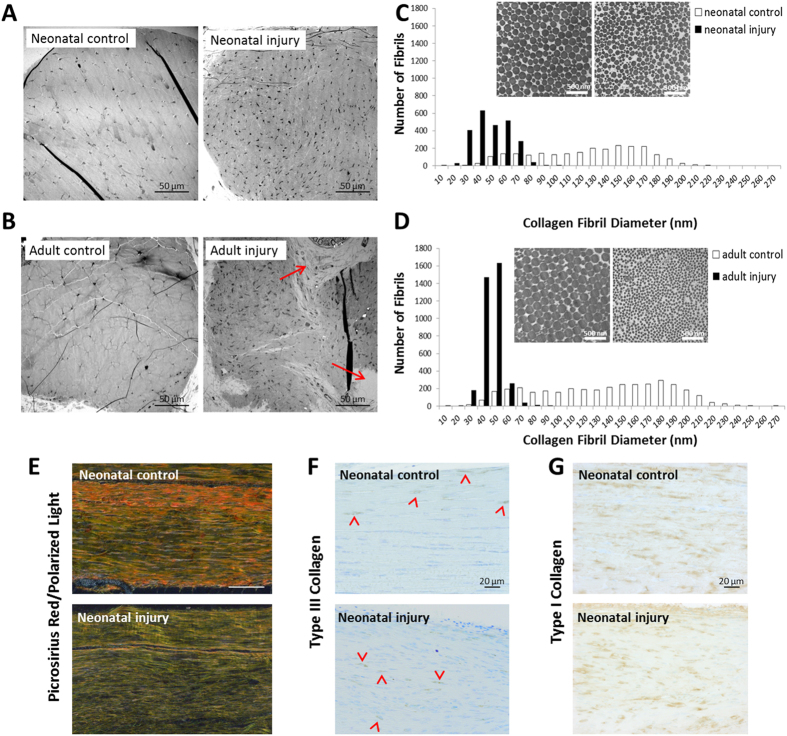
Collagen matrix is highly organized and aligned after neonatal tendon injury. TEM imaging of (**A**) neonatal and (**B**) adult tendon cross-sections at d56. Red arrows indicate disorganized, noncollagenous matrix. Quantification of (**C**) neonatal and (**D**) adult collagen fibril diameter in control and injured tendons at d56, using high magnification TEM images (insets). Fibrils were counted from n = 3 mice/group (1200 fibrils/tendon). (**E**) Picrosirius red staining and polarized light imaging of longitudinal neonatal tendon sections at d56. Green color indicates thinner fibrils while red/orange color indicates thicker fibrils. Immunostaining for (**F**) type III and (**G**) type I collagens in longitudinal neonatal tendon sections at d56. Red arrowheads highlight positive staining in (**F**).

To determine matrix ultrastructure and organization, we used transmission electron microscopy (TEM) imaging of transverse sections through the tendon mid-substance. Neonatal tendon matrix at d56 after injury was uniformly collagenous, similar to control (although higher cellular density was observed) (Fig. 4A). In contrast, adult tendon matrix at d56 after injury was less organized, with regions of noncollagenous matrix (Fig. 4B, red arrows). Quantification of fibril diameter showed that by d56, collagen fibril diameter was smaller in injured tendon compared to respective controls. Fibril diameters in both injured tendon groups were also far more homogeneous, although fibrils in the neonatal group showed a slight shift toward fibrils of larger sizes (~3x the number of >60 nm fibrils) compared to adult injury tendons, which had a higher population of smaller sized fibrils (2x the number of <60 nm fibrils) (Fig. 4C,D). Picrosirius red staining and polarized light imaging of longitudinal plastic sections also confirmed a high degree of collagen alignment at d56. Consistent with the TEM results, the green color for injured tendon indicated abundance of smaller collagen fibrils compared to the orange/red color indicating larger diameter fibrils in the contralateral control (Fig. 4E). The homogeneous distribution of small diameter collagen fibrils in injured tendons is similar to the appearance of tendon matrix during embryonic and early postnatal stages^10^, indicating that collagen maturation is not achieved in either group even after 56 days of healing.

Since type III collagen is a known regulator of type I collagen fibrillogenesis (type I collagen fibrils in *Col3a1* null skin are much larger in size with heterogeneous distribution)^17^, we considered the possibility that the sustained shift toward small collagen fibrils after injury is due to overabundance of type III collagen. The sustained presence of type III collagen has also been previously reported in studies of adult tendon injury. We therefore immunostained for type III collagen at d56, however comparable staining was observed between control and injured tendons in the neonatal group (Fig. 4F). Immunostaining for type I collagen confirmed that the majority of collagen present in the regenerated tendon is type I (Fig. 4G). Overall, these results indicate that neonatal tendon structure is more organized after injury compared to adults, with larger collagen fibrils. However, despite improved organization, the collagen matrix ultrastructure in the neonatal group remained immature 56 days after injury, indicating some structural limitations to neonatal regeneration.

### Infiltration of αSMA expressing cells during healing

**Figure 5:**
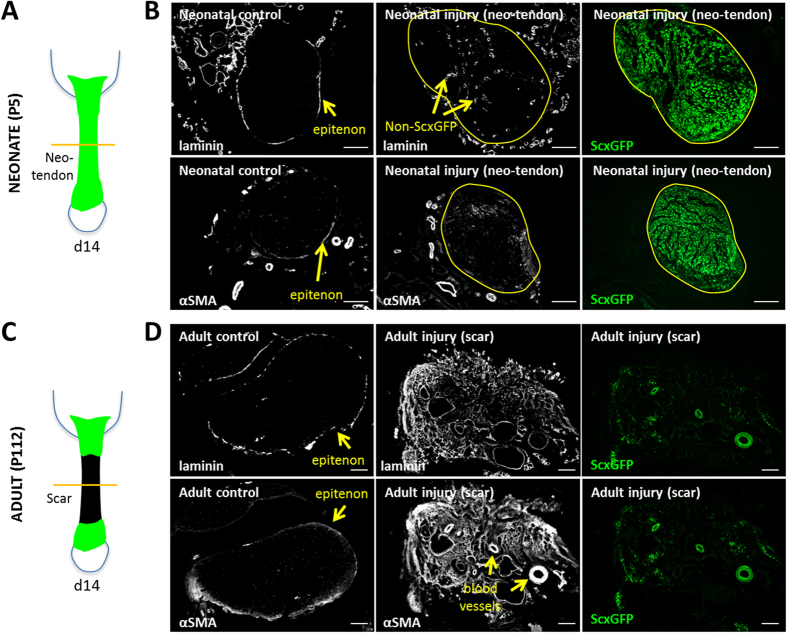
Fibrotic scar markers are transient in neonates but persist in adult injured tendon. (**A**) Schematic image and (**B**) transverse sections of neonatal *ScxGFP* hindlimb immunostained for laminin or αSMA at d14 after injury. Yellow borders delineate *ScxGFP*+ neo-tendon in (**B**). (**C**) Schematic image and (**D**) transverse sections of adult *ScxGFP* hindlimb stained for laminin or αSMA. Scalebar: 100 μm.

**Figure 6:**
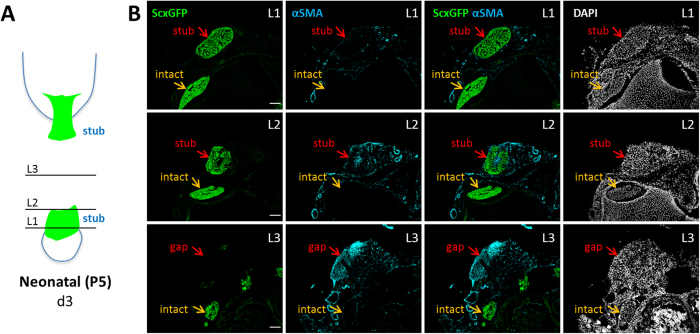
Early recruitment of αSMA+ cells into the gap space after neonatal injury. (**A**) Schematic image of transected tendon at d3 depicting section levels. (**B**) Transverse sections from *ScxGFP* limb at d3 after injury at levels L1-L3 shown in (**A**), immunostained for αSMA and counterstained with DAPI for cell nuclei. Injured Achilles tendon and adjacent non-injured tendon highlighted by red and orange arrows, respectively. Scalebar: 100 μm.

Since the epitenon (an epithelial layer surrounding tendon) has been implicated in adult tendon healing^18^,^19^, we immunostained for two markers associated with the epitenon (laminin and αSMA) and confirmed positive epitenon staining surrounding neonatal and adult control tendons (Fig. 5A–D). Notably, tenocytes were not labeled by either marker (Supplementary Fig. S2). At d14, we observed intense staining of both markers within the adult tendon scar, with only limited staining in the neonatal neo-tendon (Fig. 5B,D). Immunostaining for αSMA also revealed the presence of numerous blood vessels populating the adult scar tissue, while αSMA+ blood vessels were never observed in the neo-tendon. Interestingly, immunostaining at d3 after neonatal injury showed abundant αSMA+ cells within the gap space and infiltrating the tendon stubs near the cut site; however, at this timepoint the αSMA+ cells were completely *ScxGFP*-negative, suggesting αSMA+ cells are a separate population of cells transiently recruited after neonatal tendon injury (Fig. 6A,B).

### Lineage tracing reveals activation and recruitment of tenocytes after neonatal injury

**Figure 7:**
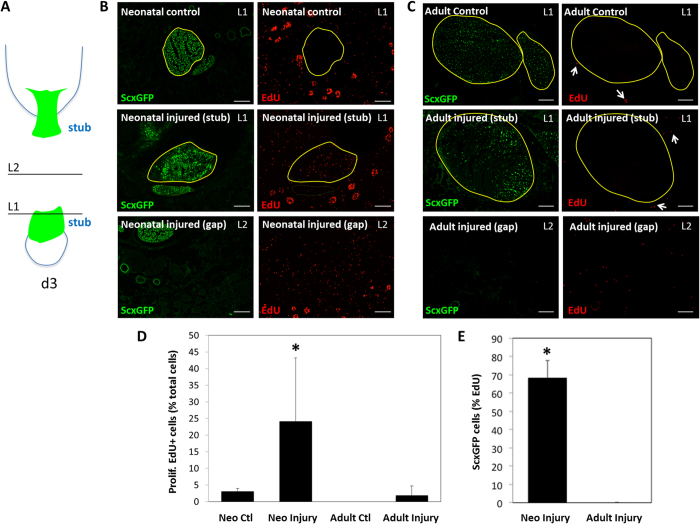
Intense tenocyte proliferation after neonatal injury while adult tenocytes remain quiescent. (**A**) Schematic image of transected tendon at d3 after injury depicting levels L1 and L2 through which (**B**,**C**) transverse sections were taken. (**B**) EdU labeling of proliferating cells in control and injured ScxGFP limbs at d3 after neonatal injury. (**C**) EdU labeling of proliferating cells (white arrows) in control and injured *ScxGFP* limbs at d3 after adult injury. Yellow borders delineate neonatal and adult tendon stubs in (**B**,**C)**. (**D**,**E**) Quantification of proliferating cells after neonatal and adult injury. Data are plotted as % EdU+ cells relative to total cells within tendon stub and % *ScxGFP*+ /EdU+ cells relative to total EdU+ cells within tendon stub. *Indicates signficant differences relative to all other groups (p < 0.05). Scalebar: 100 μm.

The transient presence of αSMA+ cells between d3 and d14 in neonates initially suggested that αSMA cells may represent a progenitor population recruited from the epitenon. However, we also detected abundant proliferation of *ScxGFP*+ cells by EdU, that was largely concentrated near the cut ends at d3, suggesting that intrinsic tenocytes may play a role in neonatal healing (Fig. 7A,B). In contrast, *ScxGFP*+ tenocytes in adults remained quiescent after injury; EdU labeling detected only *ScxGFP*- cells extrinsic to the tendon and within the gap space at d3 (Fig. 7C). Quantification of cell proliferation within the neonatal tendon stub confirmed that 24% of cells (labeled by DAPI) were proliferating after injury, compared to only 3% in the control tendon. Of the proliferating EdU+ cells in the injured tendon, the majority (~65%) were *ScxGFP*+ in neonates while 0% of proliferating cells in adults were *ScxGFP*+ (Fig. 7D,E).

**Figure 8:**
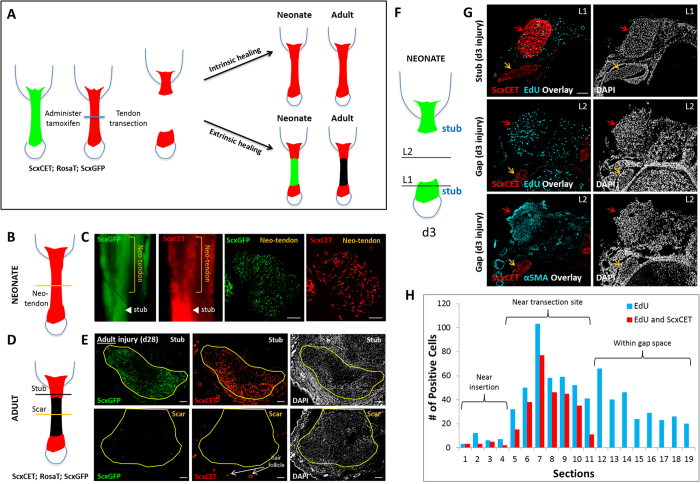
Neonatal tendon healing is driven by recruitment of intrinsic tenocytes. (**A**) Potential models of tendon healing. Neonatal: neo-tendon formation and tenogenic differentiation is driven by intrinsic tenocytes OR extrinsic cells. Adult: scar formation originates from intrinsic tenocytes OR extrinsic cells. (**B**) Schematic image and (**C**) whole mount and transverse sections of *Scx*^*CreERT2*^*/RosaT/ScxGFP* neonatal limbs at d28 after injury. Tenocytes were labeled by tamoxifen injection prior to injury. (**D**) Schematic image and (**E**) transverse sections of *Scx*^*CreERT2*^*/RosaT/ScxGFP* adult limbs at d28 after injury. Tenocytes were labeled by tamoxifen injection prior to injury. DAPI staining shows presence of numerous cells. (**F**) Schematic image of transected neonatal tendon at d3 after injury depicting levels L1 and L2 through which (**G**) transverse sections were taken. (**G**) EdU detection of proliferating cells in tamoxifen-labeled control and injured *Scx*^*CreERT2*^*/RosaT/ScxGFP* limbs at d3. Injured Achilles tendon and adjacent non-injured tendon highlighted by red and orange arrows, respectively. (**H**) Quantification of EdU+ and EdU+/*Scx*^*lin*^ cells in alternate serial transverse sections from insertion into gap space at d3 after neonatal injury from a representative tendon. Scalebar: 100 μm.

To determine the source of the cells driving neonatal vs adult tendon healing, we next used an inducible *Scx*^*CreERT2*^ line combined with the *Rosa26-TdTomato (RosaT*) Cre reporter to label tenocytes with tamoxifen prior to injury. If healing is driven by intrinsic tenocytes, we would expect to find *RosaT*+ cells within the neo-tendon or scar region; conversely, if healing is driven by extrinsic cells, *RosaT*+ cells would not be recruited into the defect (Fig. 8A). At d14 and d28 after neonatal injury, analysis of whole mount limbs and transverse sections showed that the *ScxGFP*+ neo-tendon was strongly composed of *RosaT*+ cells, suggesting that the neo-tendon is derived from *Scx-lineage (Scx*^*lin*^) tenoyctes that are recruited from the original tendon stubs (Fig. 8B,C). In adults, we found no *RosaT*+ cells in the *ScxGFP*-neg scar at d14 or d28, despite the presence of numerous cells in this region (indicated by DAPI stained nuclei) (Fig. 8D,E). EdU labeling of *Scx*^*CreERT2*^; *RosaT*; *ScxGFP* injured limbs at d3 revealed abundant proliferating *Scx*^*lin*^ cells within the injured tendon stub (red arrow), however the proliferating cells within the gap space were not *Scx*^*lin*^(Fig. 8F,G). Adjacent non-injured tendons showed minimal or no proliferation (orange arrows). Systematic analysis and quantification of alternating transverse sections from the skeletal insertion to the gap space indicated enhanced cell proliferation in tendon sections near the transection site (Fig. 8G,H). *Scx*^*lin*^ cells were localized completely to the tendon stubs and *RosaT*+ cells were not observed in the gap. Immunostaining for αSMA also showed that the αSMA+ cells present at d3 were not derived from *Scx*^*lin*^ tenocytes (Fig. 8G).

Finally, we considered the possibility that cell survival in neonatal tendons after injury may enable tenocyte recruitment, since it was previously found that adult tenocytes near the transection site undergo apoptosis shortly after injury^20^. However, Tunel staining for apoptotic cells 2 hours after injury showed intense positive staining that was localized to regions immediately adjacent to the cut site (Supplementary Fig. S3), similar to previous reports of adult injured tendons. Collectively, our results suggest that neonatal healing is mediated by transient infiltration of αSMA+ cells, followed by proliferation and recruitment of intrinsic *Scx*^*lin*^ tenocytes. In contrast, adult healing is solely mediated by extrinsic cells that persist to form a permanent scar.

### Adult tendon undergoes aberrant differentiation toward cartilage and bone after injury

**Figure 9:**
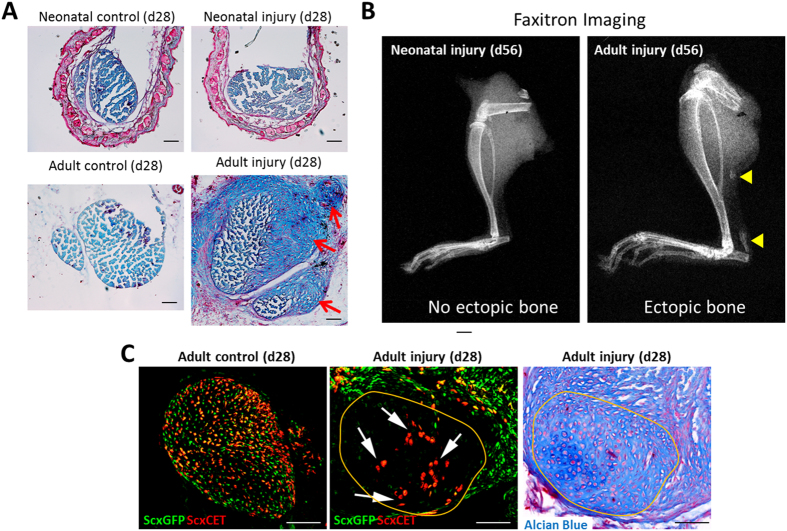
Transdifferentiation of adult tenocytes into chondrocytes during adult tendon healing. (**A**) Alcian blue cartilage staining of transverse tendon sections from control and injured limbs at d28 after neonatal or adult injury. Red arrows highlight ectopic cartilage. (**B**) Radiographs of whole limbs detect ectopic ossification at d56 in adult tendon stubs (yellow triangles). (**C**) Transverse sections through *Scx*^*CreERT2*^-labeled tendons show contribution of labeled (red) cells in regions corresponding to ectopic cartilage (white arrows). Alcian Blue staining of adjacent section confirms cartilage deposition and morphology. Scalebar: 100 μm.

Since aberrant differentiation has been reported in adult tendons after transection, we used Alcian Blue staining to visualize cartilage. Intense cartilage-like staining was observed for the adult injured tendon near the transection site at d28, while Alcian Blue staining in neonatal injured tendon was similar to controls (Fig. 9A). Radiographs showed ectopic bone formation in adult injured tendons at d56 (in ~60% of tendons), localized specifically within the tendon stubs (Fig. 9B, yellow triangles). In contrast, bone was never observed within the neonatal injury tendons at this timepoint. Since adult tenocytes appeared to be activated in response to injury (indicated by upregulated *ScxGFP* expression, Fig. 1) but were not recruited, we hypothesized that adult cells may undergo aberrant differentiation toward chondrogenesis at the expense of tenogenesis. To determine whether the ectopic cartilage in adult injured tendon was derived from *Scx*^*lin*^ cells, we again used *Scx*^*CreERT2*^ to label adult tenocytes prior to injury and harvested limbs at d28. The chondrogenic mass was readily identified by the cobblestone cell morphology and absence of *ScxGFP* expression; this was also confirmed by Alcian Blue staining of alternate adjacent sections. Inspection of *Scx*^*lin*^ cells within the sections showed that *Scx*^*lin*^ cells were incorporated within the chondrogenic mass, although numerous non-*Scx*^*lin*^ cells were also observed (Fig. 9C). This data suggests that aberrant differentiation is a feature of adult tendon healing but not neonatal tendon healing, further highlighting the divergent mechanisms that regulate neonatal vs adult tendon healing.

## Discussion

**Figure 10:**
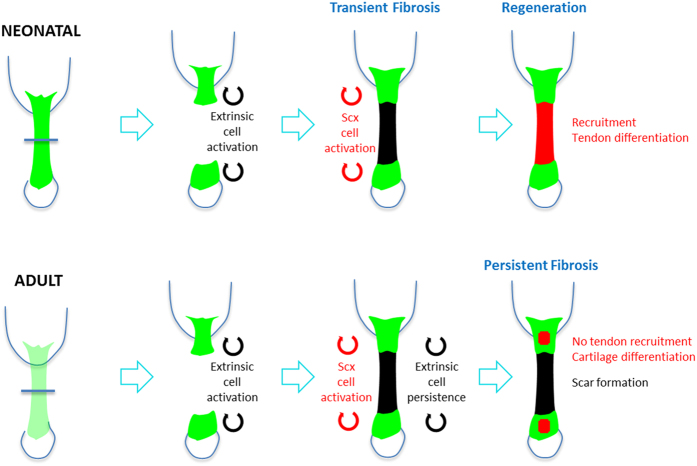
Cellular mechanism of neonatal regenerative and adult fibrotic tendon healing. Neonatal regenerative tendon healing is driven by early and transient recruitment of an extrinsic αSMA cell population, followed by tenocyte recruitment, differentiation, and restoration of function. In contrast, adult tenocytes are activated but not recruited. Instead, tenocytes contribute to ectopic cartilage formation in tendon stubs. In the absence of tenogenic cell recruitment, αSMA cells persist to form a permanent scar with impaired functional properties.

To date, only a few tissues have been tested for regenerative healing in neonates; for the heart and digit tips, neonatal regeneration is driven by lineage-restricted cells, while regeneration of cochlear hair cells also depends on transdifferentiation of a neighboring supporting cell type^11^,^12^,^13^,^14^. Using our neonatal injury model, lineage tracing showed that tendon regeneration is driven by tenocyte proliferation and recruitment from the original tendon stubs. In contrast, adult tenocytes are quiescent after injury and do not undergo recruitment; in the absence of tenogenic cells, adult fibrotic healing is mediated by αSMA-expressing cells that persist to form a permanent scar (Fig. 10). Our results suggest that the intrinsic potential of neonatal tenocytes (which are still mitotically active at this stage and relatively immature) is likely very different compared to adult tenocytes, which are post-mitotic cells. It can be difficult to conceptually separate development vs regeneration at this stage since the tissue is still undergoing dramatic growth (although the key events of cell specification, differentiation, and patterning happen during embryonic stages)^21^,^22^. And indeed, regeneration often recapitulates many aspects of tissue development. For example, in the case of regenerative organisms (such as adult salamanders) or tissues (such as bone), it can be argued that the regenerative process recapitulates aspects of development during the course of healing^23^,^24^. Unlike development however, the cells driving tissue regeneration are more restricted in their lineage potential and the local inflammatory environment plays a critical role^13^,^25^,^26^.

Although we identified tenocyte recruitment as a key driver of neonatal healing, we cannot yet exclude the possibility that a progenitor/stem cell type may also be activated after injury. Indeed, it was suggested that tendon stem cells may reside within the tendon proper^27^; therefore, recruited cells may represent a specialized sub-population of tenocytes with ‘stem’ potential. Other studies however, suggest that tendon stem cells may reside within the epitenon/paratenon and express αSMA^19^,^28^,^29^,^30^. While we propose here that αSMA+ cells initially form a transient fibrotic tissue after neonatal tendon injury, we also identified a small subset of αSMA+ cells within the neo-tendon that turned on *ScxGFP* at later stages. Lineage tracing using the transgenic *αSMA*^*CreERT2*^ to fate map this population was attempted, but the extrinsic αSMA cell population could not be separated from neonatal tenocytes at these stages due to extensive Cre-labeling of tenocytes (unpublished data). This labeling likely does not reflect endogenous expression since αSMA immunostaining does not label tenocytes at any postnatal stage. Although αSMA staining was observed in the epitenon, it is also possible that these cells may be derived from multiple external sources^31^. Elucidating the roles of this intriguing cell population and identifying other potential cell types involved in regenerative and fibrotic tendon healing will be the focus of future studies.

One interesting feature of adult tendon healing is the strong re-activation of *ScxGFP* expression after injury and aberrant differentiation toward cartilage and bone. The specific localization of *ScxGFP* re-expression and aberrant cartilage formation to the tendon stubs suggest that these events may be interrelated, and indeed we found *Scx*^*lin*^ cells within the cartilage masses, consistent with two recent studies that also show a contribution of tenocytes to heterotopic ossification, either in the context of injury or via constitutive activation of the BMP receptor, ACVR1^32^,^33^. During embryonic development, an early pool of bipotent *Scx*+ /*Sox9*+ progenitors give rise to either the cartilage or tendon components of the skeletal enthesis^34^,^35^. We speculate that re-activation of *ScxGFP* after injury may indicate tenocyte reversion toward a progenitor-like phenotype; adult tenocytes may subsequently undergo chondrogenesis in response to abnormal mechanical loading (or rather, unloading) associated with transection injury or in response to inflammatory cues. Sensitivity of tenocytes to their mechanical environment has been shown in several studies. For example, the application of compressive forces in tendon can induce fibrocartilaginous phenotypes, which is lost when the mechanical stimulation is removed^36^,^37^. Inflammation can also trigger heterotopic endochondral ossification, as established by several studies of traumatic or neurogenic injuries and congenital diseases^38^,^39^,^40^. In addition, a recent study showed that the tendon transcription factor *Mkx* may also function to inhibit chondrogenic differentiation in tenocytes;^41^ our study showed downregulation of *Mkx* expression after adult tendon injury, which may permit cartilage differentiation at the expense of tenogenesis. Although our lineage tracing results showed a clear contribution of *Scx*^*lin*^ tenocytes to ectopic cartilage, we also observed a number of non-labeled cells. This may be due to incomplete recombination of tenocytes or may also indicate infiltration of extrinsic cells to help form ectopic cartilage. If extrinsic cells are recruited to the stubs, the specific localization of the ectopic cartilage to regions near the cut site suggests that chondrogenic differentiation of extrinsic cells is likely driven by signals from the injured tendon itself or immune cells that home to those regions. Overall, our data indicates that aberrant differentiation of tenocytes toward cartilage at the expense of tenogenic recruitment may be one of the key mechanisms underlying poor adult tendon healing. Identifying the local mechanical or molecular signals that inhibit tendon cell recruitment into the defect may enable targeted therapies to induce adult tendon regeneration.

The signaling pathways underlying regenerative and fibrotic tendon healing have yet to be elucidated, but one attractive candidate is the TGFβ/BMP family. During development, TGFβ signaling is essential for tendon formation and it is well established that TGFβ can induce tendon markers in cell culture. Interestingly, TGFβ can also drive chondrogenic differentiation and injection of TGFβ ligands has been used to induce cartilage deposits in tendon, suggesting a potential role for TGFβ in ectopic cartilage formation after tendon injury as well^42^,^43^,^44^. In the context of injury, TGFβ has also been implicated as a potent inducer of fibrosis. While BMP signaling generally inhibits tendon during development (while inducing cartilage), select members of the BMP family (BMPs 12, 13, and 14) may drive tendon differentiation^45^,^46^,^47^,^48^. Future studies will therefore elucidate the specific activities of TGFβ, BMP, and their downstream signaling and interactions in tendon regeneration, fibrosis, and heterotopic ossification.

Neonates demonstrated full recovery of all functional properties tested, including gait and tensile properties. This is in marked contrast with adult tendons, which remain functionally impaired after injury, consistent with numerous studies in the literature^49^,^50^,^51^,^52^,^53^. Since function is the gold standard by which tendon healing is frequently assessed, our finding of improved neonatal tendon function is exciting and comparable to other existing regenerative tendon models, such as MRL/MpJ (and related mouse strains) and fetal sheep, which also recover functional properties after injury^54^,^55^,^56^,^57^,^58^. Although MRL/MpJ and related strains are useful models of adult regeneration in specific contexts, a few recent studies in non-tendon tissues suggest that MRL/MpJ may exhibit accelerated wound closure via excessive scar formation rather than true regeneration^59^,^60^,^61^,^62^. The fetal model on the other hand is technically challenging in mice given the small size and inaccessibility of mouse embryos. Our neonatal mouse model overcomes these limitations since its regenerative potential is not restricted to any particular strain; thus, we can test gene function and cellular mechanisms using the wide range of genetic tools that have already been generated for mouse and directly compare against adult non-regenerative tendon healing within the same genetic background.

Although our neonatal tendon regeneration model captures key aspects of tendon differentiation, maturation of the collagen matrix is not restored two months after injury, despite functional recovery. Our results suggest that although *Mkx* is necessary for collagen maturation^9^,^10^, it may not be sufficient (since we do find significant upregulation of *Mkx* after neonatal injury) or may not be expressed to sufficient levels. Additional factors may be required for full maturation of collagen structure; these factors may include type V, XII, or XIV collagens or non-collagenous matrix molecules such as decorin and fibromodulin^63^,^64^,^65^. An additional limitation includes the choice of complete Achilles tendon transection injury without repair, which is less representative of the clinical scenario. Tendon ruptures are typically preceded by accumulated local damage caused by overuse (due to sports activities or age), although in the case of knife wounds, laceration of healthy tendons can also occur^66^. Several overuse models have been developed for mouse tendon^67^,^68^, however these models are not readily adapted to the neonatal mouse and the narrow time window required for regenerative healing. The advantages of full transection is experimental feasibility during these early postnatal stages, high reproducibility, and the large number of studies using this model for adult tendon healing in the literature. For studies of basic tendon biology, our neonatal model for tendon regeneration holds exciting promise for establishing important cell and molecular events that regulate tendon differentiation, with the potential to inform adult healing and the development of new therapies.

## Methods

### Mice

Existing mouse lines were used in these studies: *ScxGFP* tendon reporter^69^, *Scx*^*CreERT2*^ (generated by Dr. Ronen Schweitzer), and the *Ai14 Rosa26-TdTomato* Cre reporter^70^. Lineage tracing was carried out by tamoxifen administration prior to injury. Tamoxifen in corn oil was given to neonates by gavage (1.25 mg/pup for 2 consecutive days, followed by 2 days rest) and to adults by daily intraperitoneal injections (100 mg/kg wt for 3 consecutive days followed by 2 days rest). EdU was given at 0.05 mg 2 hr prior to harvest to label proliferating cells. All animal procedures were approved by the Institutional Animal Care and Use Committee and Icahn School of Medicine at Mount Sinai and are consistent with animal care guidelines.

### Tendon injury model

For all studies, neonatal (P5) and adult (4–5 month old) mice were anesthetized by hypothermia or isofluorane inhalation, respectively. A small incision was made in the skin of right hindlimbs to expose the Achilles tendon, followed by complete transection of the Achilles tendon at the midsubstance. The left contralateral limb was used as controls. After injury, the skin was closed using Prolene sutures and animals returned to full cage activity. Male and female mice were distributed evenly between groups.

### RNA isolation, reverse transcription, and qRT-PCR

Total RNAs were extracted from contralateral control or injured Achilles tendons after neonatal or adult injury using Trizol/chloroform and quantified using NanoDrop 2000. Reverse transcription was carried out using SuperScript VILO Master Mix and qRT-PCR performed using SYBR Green PCR Master Mix (Thermo Fisher). The primer sequences used are listed in Supplementary Table S2. RNA samples were prepared from 5–7 independent samples and ran in triplicate.

### Functional gait analysis

DigiGait Imaging System and software (Mouse Specifics Inc., Quincy, MA) were used to analyze functional gait recovery following injury. Without pretraining, mice were gaited at 5 cm/s for 3–4 s on a transparent treadmill and a high-speed digital camera used to capture the position of each paw. Footage was analyzed using the Digigait Analysis Software (Digigait 12.4). Measurements from the hindlimbs were used to calculate %SwingStride, %BrakeStride and %PropelStride (all parameters were normalized to Stride Length to account for differences in animal size due to sex or age). Injured and non-injured age-matched mice were used to establish gait parameters associated with injury and healing (n = 5–8 per group).

### Biomechanical testing and analysis

Mechanical testing of mouse Achilles tendons was performed using custom grips to clamp the calcaneus bone and Achilles tendon. The tissue was then immersed in a PBS bath at room temperature and preloaded to 0.05 N for ~1 min followed by ramp to failure at 1%/s. The maximum force, stiffness and elongation at yield were recorded for both injured and uninjured tendons. Material properties were not calculated since cross-sectional area could not be accurately measured in tissues of this size. Mechanical testing was carried out for n = 10 independent samples per group.

### Transmission electron microscopy

Whole mouse hindlimbs were fixed in 1.5% glutaraldehyde/1.5% paraformaldehyde (Tousimis Research Corporation) in Dulbecco’s serum-free media (SFM) containing 0.05% tannic acid, followed by an extensive rinse in SFM, then post-fixation in 1% OsO_4_ Samples were washed in SFM then dehydrated in a graded series of ethanol to 100%, rinsed in propylene oxide, and infiltrated in Spurrs epoxy. Samples were polymerized at 70 C over 18 h. TEM images of transverse sections were collected at several magnifications to enable morphological visualization of the collagen fibrils and gross tendon appearance. Collagen fibril diameters were determined using Image J software (NIH); six representative images were analyzed for each tendon (n = 3 independent samples per group) and a total of 1200 fibrils analyzed per sample.

### Whole mount fluorescence imaging

Whole mount imaging was carried out for whole limbs fixed in 4% paraformaldehyde with skin removed. Images were acquired using a Leica stereomicroscope with fluorescence capabilities (Leica M165FC).

### Histology and immunofluorescence

For fluorescence imaging, immunofluorescence, and *in situ* hybridization of sections, hindlimbs were fixed in 4% paraformaldehyde and frozen in OCT medium. Alternating transverse cryosections (12 μm) were collected across the length of the limb to capture the trajectory of the Achilles tendon from skeletal insertion to muscle. Immunostaining was carried out using antibodies against laminin (Sigma) and α-smooth muscle actin (Sigma) with Cy3 or Cy5 secondary detection (Jackson ImmunoResearch), with DAPI counterstaining to visualize cell nuclei. EdU and Tunel assays were performed using the Click it EdU (Life Technologies) and *In Situ* Cell Death Detection kits (Roche), respectively, according to manufacturer’s instructions.

For analysis of longitudinal sections, hindlimbs were fixed in zinc formalin, dehydrated, and infiltrated with methacrylate monomer and embedded. Plastic sections were then acquired at 6 μm, stained with Picrosirius Red, and imaged with polarized light to visualize collagen alignment. Additional sections were immunostained for type I and III collagens (Abcam) with DAB Chromagen secondary detection (Vector Laboratories) and counterstained with Toluidine Blue.

All images were acquired using Zeiss Axio Imager microscope; an Apotome was used for optical sectioning of fluorescent images.

### Statistics

All quantitative results are presented as mean ± standard deviation. For qRT-PCR analysis, statistics were carried out using two way ANOVA with Tukey’s posthoc testing (independent variables of time and injury condition, Systat Software). Cell proliferation quantification was analyzed using one way ANOVA. All other quantitative analyses were carried out using paired Students t-tests between control and injured samples. Significant outliers were detected and discarded using the Grubbs’ test (Graphpad, α = 0.05).

## Additional Information

**How to cite this article**: Howell, K. *et al*. Novel Model of Tendon Regeneration Reveals Distinct Cell Mechanisms Underlying Regenerative and Fibrotic Tendon Healing. *Sci. Rep.* **7**, 45238; doi: 10.1038/srep45238 (2017).

**Publisher's note:** Springer Nature remains neutral with regard to jurisdictional claims in published maps and institutional affiliations.

## Supplementary Material

Supplementary Figures and Tables

Supplementary Video 1

Supplementary Video 2

Supplementary Video 3

Supplementary Video 4
