# Supplementary material for: Novel Model of Tendon Regeneration Reveals Distinct Cell Mechanisms Underlying Regenerative and Fibrotic Tendon Healing
Source: Sci Rep. 2017 Mar 23;7:45238. doi: 10.1038/srep45238 (PMC5362908; doi:10.1038/srep45238)
Supplement: Supplementary Figures and Tables [file srep45238-s1.pdf]

NOVEL MODEL OF TENDON REGENERATION REVEALS DISTINCT CELL MECHANISMS UNDERLYING REGENERATIVE AND FIBROTIC TENDON HEALING

Kristen Howell[1], Chun Chien[1], Rebecca Bell[2], Damien Laudier[1], Sara F. Tufa[3], Douglas R. Keene[3], Nelly Andarawis-Puri[2], Alice H. Huang[1]

[1] Dept. of Orthopaedics
Icahn School of Medicine at Mount Sinai
New York, NY 10029 USA

[2] Dept. of Mechanical and Aerospace Engineering
Cornell University
Ithaca, NY 14853 USA

[3] Micro-Imaging Center
Shriners Hospital for Children
Portland, OR 97209, USA

Supplementary Figures and Tables

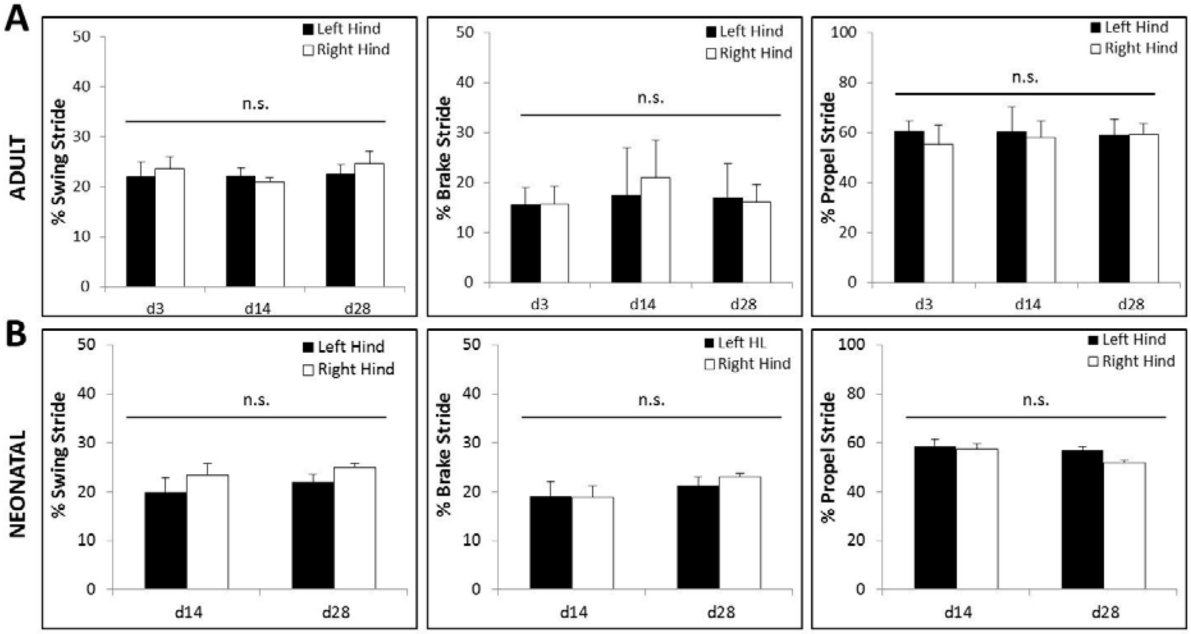

Figure S1: Quantitative gait is not affected in non-injured animals at any timepoint. Gait analysis for age-matched (**A**) adult and (**B**) neonatal mice do not show differences between left and right non-injured hindlimbs at any timepoint. n=5; n.s. indicates no significance, p>0.1 for all comparisons.

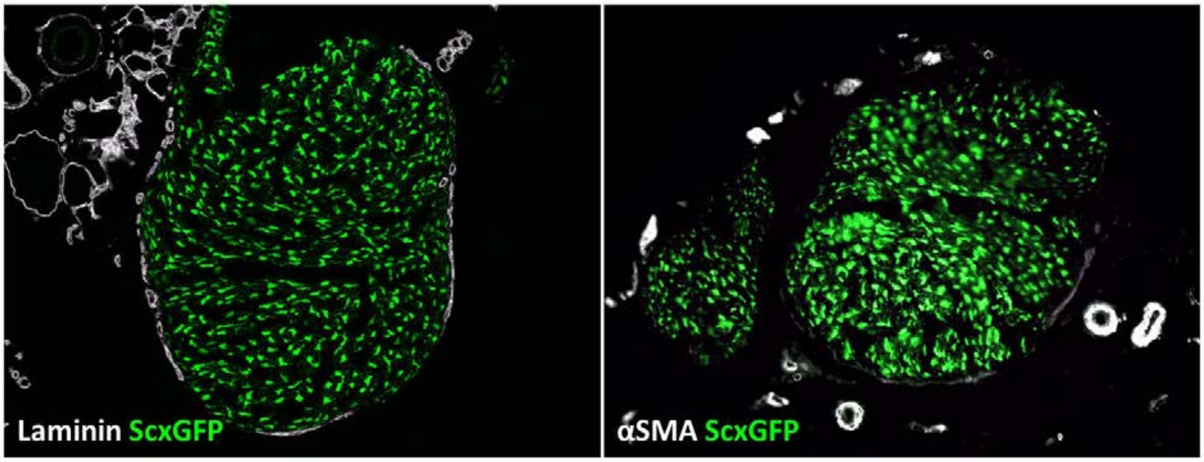

Figure S2: Laminin and αSMA immunostaining is restricted to the epitenon. ScxGFP expressing tenocytes do not overlap with laminin or αSMA immunostaining.

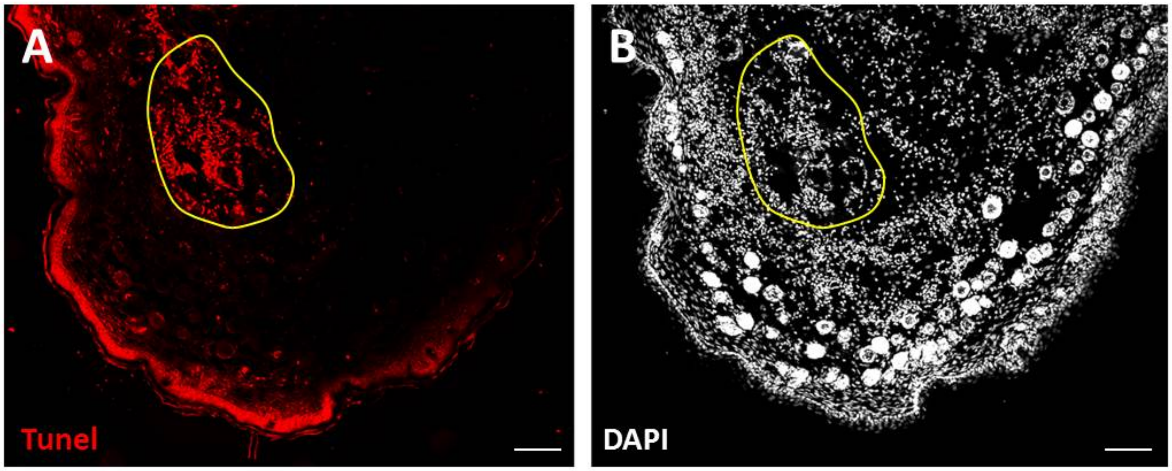

Figure S3: Neonatal tenocytes adjacent to the cut site undergo apoptosis following tendon transection. (**A**) Tunel and (**B**) DAPI staining of transverse section across Achilles tendon adjacent to the transection site 2 hours after initial injury.

Supplementary Table 1: Tendon genes are unchanged or downregulated after adult tendon injury. Real time qPCR gene expression of Scx, Tnmd, and Mkx in control and injured tendon from d3 through d28. * indicates significant difference relative to control within timepoint ($p<0.05$), # indicates trend toward significant difference relative to control within timepoint ($p<0.1$). n=5-6.

	Scx		Tnmd		Mkx	
**Time**	**Control**	**Injury**	**Control**	**Injury**	**Control**	**Injury**
d3	3.60 ± 2.66	1.50 ± 4.04	4.31 ± 2.82	1.84 ± 8.59	1.08 ± 0.75	0.23 ± 0.05
d7	4.16 ± 3.00	12.97 ± 11.04	10.54 ± 5.02	24.50 ± 22.06	3.14 ± 1.86	0.67 ± 0.28
d14	26.05 ± 24.22	6.17 ± 3.99$^{\#}$	133.47 ± 142.13	136.83 ± 67.61	28.71 ± 30.31	1.29 ± 0.35*
d28	2.69 ± 3.03	3.44 ± 1.62	10.11 ± 10.40	64.12 ± 30.56	1.27 ± 1.56	1.44 ± 0.69

Supplementary Table 2: Primer sequences used for real time qPCR generated using NCBI Primer-BLAST and validated against RNA derived from E14.5 embryos or P0 pups.

Gene	Fwd Primer	Rev Primer
Scx	CGTCTTTCTGTCACGGTCTTTGCTC	CTTTCTTCCACAGCGGTCGTGC
Tnmd	GGGCTGTCACATTCTAAATGCAG	TTCTTCTTCTCGCCGTTGCT
Mkx	CGTGACAACCCGTACCCTAC	TTTGACACCTGCACTAGCGT
Col1a1	ACGCCATCAAGGTCTACTGC	ACTCGAACGGGAATCCATCG
Col2a1	ATCTTGCCGCATCTGTGTGT	GGCCCTAATTTTCCACTGGC
Col3a1	CCAGTGGCCATAATGGGGAA	ATCTCGACCTGGCTGACCAT
Ocn	ATTTAGGACCTGTGCTGCCC	GCAGAGAGAGAGGACAGGGA
Cfd	CACAGCTCCGTGTACTTCGT	ACTTGCACGGAAGCCATGTA
αSMA	GTACCCAGGCATTGCTGACA	GAGGCGCTGATCCACAAAAC
Fb1	ATGAGAAGCCTGGATCCCCT	GGAAGGGTAACCAGTTGGGG
GAPDH	TGATGACATCAAGAAGGTGGTGAAG	TCCTTGGAGGCCATGTAGGCCAT

Supplementary Video Legends

Supplementary Video 1: Control neonatal pup (noninjured) at P8

Supplementary Video 2: Control neonatal pup (noninjured) at P19

Supplementary Video 3: Injured neonatal pup 3 days after injury (P8)

Supplementary Video 4: Injured neonatal pup 14 days after injury (P19)
